# Anaemia and cerebrospinal fluid biomarkers of Alzheimer’s pathology in cognitively normal elders: the CABLE study

**DOI:** 10.1186/s12883-021-02487-z

**Published:** 2021-11-19

**Authors:** Xin-Yu Yang, Xiao-He Hou, Yan-Lin Bi, Hao Hu, Xi-Peng Cao, Lan Tan, Jiu-Long Yang, Jin-Tai Yu

**Affiliations:** 1 Department of Neurology, Qingdao Municipal Hospital, Dalian Medical University, Dalian, China; 2 Department of Neurology, Qingdao Municipal Hospital, Qingdao University, Qingdao, China; 3 Department of Anesthesiology, Qingdao Municipal Hospital, Qingdao University, Qingdao, China; 4 Clinical Research Center, Qingdao Municipal Hospital, Qingdao University, Qingdao, China; 5 Qingdao Municipal Hospital, Qingdao University, No.5 Donghai Middle Road, Qingdao, China; 6 Department of Neurology and Institute of Neurology, Huashan Hospital, Shanghai Medical College, Fudan University, No. 12 Wulumuqi Road, Shanghai, China

**Keywords:** Anaemia, Severity, Alzheimer’s disease, Cerebrospinal fluid, Biomarkers

## Abstract

**Background:**

Anaemia has been reported to be associated with cognitive decline and Alzheimer’s disease (AD), but the associations between anaemia and cerebrospinal fluid (CSF) AD biomarkers are still unknown. This study aimed to investigate the associations between anaemia and CSF AD biomarkers.

**Methods:**

Participants were included from the Chinese Alzheimer’s Biomarker and LifestylE (CABLE) study. The associations of anaemia and its severity with CSF AD biomarkers including β-amyloid 1–42 (Aβ42), total tau (t-tau) and phosphorylated tau (p-tau) were analysed by multiple linear regression models. Adjusted for age, gender, educational levels, *APOE* ε4 alleles, comorbidities (history of coronary heart disease, history of stroke, hypertension, diabetes mellitus, dyslipidaemia) and glomerular filtration rate.

**Results:**

A total of 646 cognitively normal older adults, consisting of 117 anaemia patients and 529 non-anaemia individuals, were included in this study. Anaemia patients had lower levels of CSF Aβ42 than individuals without anaemia (*p* = 0.035). Besides, participants with more severe anaemia had lower CSF Aβ42 levels (*p* = 0.045). No significant association of anaemia with CSF t-tau and p-tau levels was found.

**Conclusion:**

Cross-sectionally, anaemia was associated with lower CSF Aβ42 levels. These findings consolidated the causal close relationship between anaemia and AD.

**Supplementary Information:**

The online version contains supplementary material available at 10.1186/s12883-021-02487-z.

## Background

As a very common disease in elders, anaemia has a prevalence of 17% in individuals aged 65 or older [1]. Anaemia has been associated with increased morbidity and mortality, and it is responsible for about 8% of nonfatal health loss [2]. In addition, anaemia has been considered as a risk factor for cognitive decline and Alzheimer’s disease (AD) [3, 4]. Nonetheless, the association between anaemia and AD is still controversial.

The formation of extracellular amyloid plaques and intracellular neurofibrillary tangles is the typical pathological characteristic of AD, which can be reflected by alterations in cerebrospinal fluid (CSF) biomarkers including reduction of β-amyloid 1–42 (Aβ42), and increased total tau (t-tau) [5]. These changes may start several years before a diagnosis of dementia can be made [6]. In addition, the 2018 National Institute on Aging-Alzheimer’s Association (NIA-AA) research framework proposed a biomarker-based definition of AD, which highlighted the importance of biomarkers in the diagnosis of AD [7]. Previous studies of association between anemia and AD are that they may have relied on clinical definitions of AD, but clinical definitions are not as consistent with AD neuropathology as like CSF AD biomarkers. These biomarkers detected in the body predate clinical symptoms by many years, or even decades [8]. Just as defined by CSF biomarkers, the purpose of our study was to determine whether there is an association between anemia and AD in a population of cognitively normal individuals, which will help us explore whether the association arises at a pre-clinical stage.

In this study, we aimed to investigate the associations between anaemia and CSF biomarkers of Alzheimer’s disease pathology in a sample of 646 cognitively intact individuals from the Chinese Alzheimer’s Biomarker and LifestylE (CABLE) study.

## Methods

### Study participants

Our study only included cognitively normal individuals from the Chinese Alzheimer’s Biomarker and LifestyLE (CABLE) study. Since 2017, CABLE is a large-scale cohort study mainly focusing on Alzheimer’s risk factors and biomarkers in Chinese Han population [9]. CABLE aims to identify genetic, environmental, and lifestyle risk factors as well as AD biomarkers in Chinese Han population. All participants in the CABLE were enrolled at Qingdao Municipal Hospital, Shandong Province, China.

All participants were Han Chinese aged between 50 to 90 years. The exclusion criteria were (1) central nervous system infection, head trauma, epilepsy, multiple sclerosis, or other major neurological disorders; (2) major psychological disorders (e.g., depression); (3) severe systemic diseases (e.g., malignant tumours, circulatory diseases such as severe heart failure and kidney failure) that may affect CSF or blood levels of AD biomarkers including Aβ and tau; (4) family history of genetic disease. All participants underwent clinical and neuropsychological assessments, and blood and CSF sample collection for the next day. Participants were required to fast for at least 8 h prior. Demographic information, AD risk factor profile, and medical history were also collected by a comprehensive questionnaire and an electronic medical record system. Participants in the study were outpatients and inpatients. We excluded participants with serious medical conditions under the guidance of a professional and experienced physician.

### Haemoglobin measurements and definition of anaemia

Haemoglobin was measured from blood samples using an automated haematology analyser in the laboratory at Qingdao Municipal Hospital. Anaemia was defined as haemoglobin concentrations < 13 g/dl for men and < 12 g/dl for women. The severity of anaemia was classified as mild (11–12.9 g/dl for men and 11–11.9 g/dl for women), moderate (8–10.9 g/dl), or severe (< 8 g/dl) according to World Health Organization criteria [10].

### CSF AD biomarker measurements

The collection and management of CSF samples conform with the international consensus on standardization of CSF research [11]. CSF was collected in 10 ml polypropylene tubes by lumbar puncture in the morning after overnight fasting and was gently mixed to avoid gradient effects. Polypropylene tubes that were used for sample collection do not absorb protein. In the case of traumatic lumbar puncture, the first 1–2 mL of CSF was discarded and sample was collected after transparency [12]. CSF sample was sent to the laboratory at room temperature within 2 h. These specimens were centrifuged at 2000 g for 10 min. CSF samples were separated and stored in an enzyme-free EP (Eppendorf) tube (AXYGEN; PCR-02-C) at − 80 °C until assay. The thaw/freezing cycle was limited not to surpass 2 times. According to the manufacturer’s recommendations, CSF concentrations of Aβ42, Aβ40, t-tau, and p-tau were determined with the ELISA kit (Innotest β-AMYLOID (1–42),β-AMYLOID (1–40), hTAU-Ag, and PHOSPHO-TAU (181p); Fujirebio, Ghent, Belgium) on the microplate reader (Thermo Scientific™ Multiskan™ MK3). All ELISA measurements were performed by experienced technicians in strict accordance with the manufacturer’s instructions. They were blinded to the clinical information. Moreover, the within-batch CV was < 5% and the inter-batch CV was < 15%.

### Covariates and cognitive assessment

Fundamental covariates included age, gender, education years, *APOE* ε4 alleles. Participants were classified as *APOE* ε4 noncarriers (participants with no copies of the *APOE* ε4 gene), and *APOE* ε4 carriers (individuals with at least one copy of the *APOE* ε4 gene). DNA was extracted from the blood samples using QIAamp DNA Blood Mini Kit (250). Restriction fragment length polymorphism (RFLP) technology was used to genotype rs7421 and rs429358 to define *APOE* ε4 alleles. Hypertension, diabetes mellitus and dyslipidemia were determined using self-report, medication use, and verification of hospital records by an adjudicator according to prescribed algorithms. History of stroke and coronary heart disease were based on self-report, clinic data, and medication use. Glomerular filtration rate was estimated using the Cockcroft-Gault equation. Cognitive function was assessed using the adopted China-Modifed Mini-Mental State Examination (CM-MMSE).

### Statistical analysis

Wilcoxon tests and chi-squared tests were used to examine continuous variables (age, educational level, cognitive scores, and glomerular filtration rate, etc.) and categorical variables (gender, *APOE* ε4 alleles, history of coronary heart disease, history of stroke, history of hypertension,history of diabetes mellitus, and history of dyslipidaemia, etc) between groups respectively. Multiple linear regression models were used to investigate the associations of anaemia with CSF AD biomarkers. Age, gender, educational levels, *APOE* ε4 alleles comorbidities (history of coronary heart disease, history of stroke, history of hypertension, history of diabetes mellitus, history of dyslipidaemia), and glomerular filtration rate were included as covariates. *P* < 0.05 was considered statistically significant. All statistical analyses were performed with R software (version 3.6.1).

## Results

### Characteristics of participants

A total of 646 cognitively normal elders were included in this study, consisting of 171 anaemia patients and 529 participants without anaemia. The characteristics of included participants were summarized in Table 1. In brief, anaemia patients were older than participants without anaemia (*p* < 0.01). There were less *APOE* ε4 carriers in anaemia group (6.0%) than non-anaemia group (18.1%). Besides, participants without anaemia had higher CM-MMSE scores than anaemia patients (*p* < 0.01). Lower glomerular filtration rate and higher incidence of stroke were also observed in those with anaemia compared with those without anaemia. There was no significant difference in incidence of coronary heart disease, hypertension, dyslipidemia between those with anaemia and those without anaemia (*p* > 0.05).

**Table 1:** Participant Characteristics

Characteristic	Anemia	Non-Anemia	***P*** value
No.	117	529	–
Age (years, mean ± SD)	66.04 ± 10.99	61.14 ± 10.03	< 0.01
Female (n, %)	56, 47.9%	208, 39.3%	0.21
*APOE* ε4 carriers (n, %)	7, 6.0%	96, 18.1%	< 0.01
Education years (years, mean ± SD)	9.25 ± 4.22	9.77 ± 4.41	0.25
CM-MMSE (mean ± SD)	27.34 ± 2.31	27.95 ± 2.07	< 0.01
Coronary heart disease (n, %)	18, 15.38%	83,12.84%	0.36
History of stroke (n, %)	7, 5.98%	16, 2.47%	< 0.01
Hypertension (n, %)	49, 41.88%	246, 38.08%	0.35
Diabetes mellitus (n, %)	24,20.68%	94,14.66%	0.04
Glomerular filtration rate, mL/min	83.93 ± 22.60	91.60 ± 15.12	< 0.01
Dyslipidemia (n, %)	3, 2.56%	24,3.71%	0.46
**CSF biomarkers**			
Aβ42 (pg/ml, mean ± SD)	145.03 ± 58.07	157.39 ± 70.32	0.19
Aβ40 (pg/ml, mean ± SD)	6231.48 ± 2410.13	6116 ± 2572.62	0.40
t-tau (pg/ml, mean ± SD)	179.76 ± 87.07	158.12 ± 67.15	0.32
p-tau (pg/ml, mean ± SD)	36.82 ± 8.13	37.18 ± 8.55	0.70
T-tau/Aβ42 ratio (mean ± SD)	1.36 ± 0.80	1.19 ± 0.61	0.01
P-tau/Aβ42 ratio (mean ± SD)	0.27 ± 0.09	0.26 ± 0.09	0.10
Aβ42/ Aβ40 ratio (mean ± SD)	0.02 ± 0.01	0.03 ± 0.24	0.04

*Abbreviations*: *CSF* Cerebrospinal fluid, *CM-MMSE* China-Modified Mini-Mental State Examination, *Aβ* β-Amyloid, *t-tau* Total tau, *p-tau* Phosphorylated tau, *HGB* Hemoglobin, *SD* Standard Deviation

### Associations between anaemia and CSF AD biomarkers

**Fig. 1:**
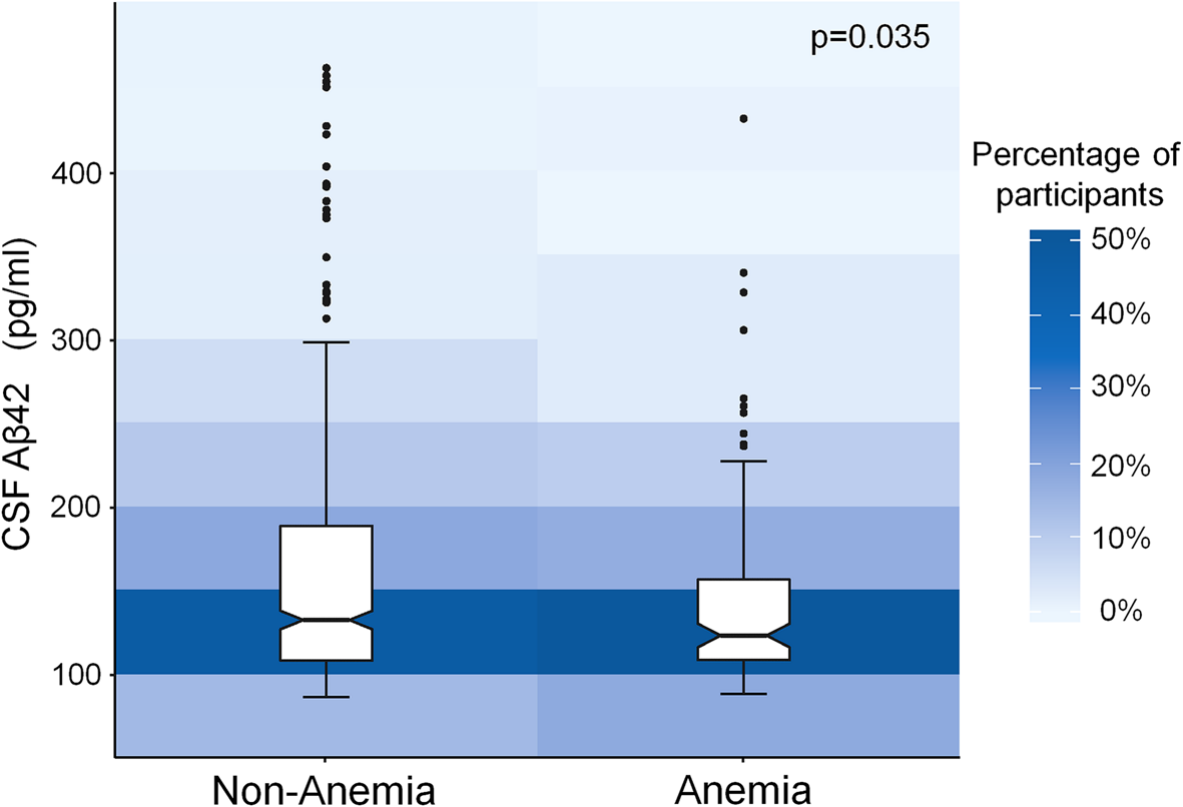
Association of anaemia with CSF Aβ42. Participants with anaemia have a lower CSF Aβ42 levels than those without anaemia (*p* = 0.035)

Firstly, we investigated the associations between anaemia and CSF biomarkers. Anaemia patients had lower levels of CSF Aβ42 (145.03 ± 58.07 pg/ml) than participants without anaemia (157.39 ± 70.32 pg/ml) after adjusting for age, gender, educational levels, *APOE* ε4 alleles, comorbidities (history of coronary heart disease, history of stroke, hypertension, diabetes mellitus, dyslipidaemia), and glomerular filtration rate (*p* = 0.035, Fig. 1). However, no significant differences were found in CSF Aβ40,t-tau, p-tau, t-tau/Aβ42, CSF p-tau/Aβ42 and Aβ42/Aβ40 levels between anaemia group and non-anaemia group (*p* = 0.799 for CSF Aβ40, *p* = 0.838 for CSF t-tau, *p* = 0.197 for CSF p-tau, *p* = 0.117 for CSF t-tau/Aβ42, *p* = 0.397 for CSF p-tau/Aβ42, *p* = 0.097 for Aβ42/Aβ40).

### Associations between the severity of anaemia and CSF AD biomarkers

**Fig. 2:**
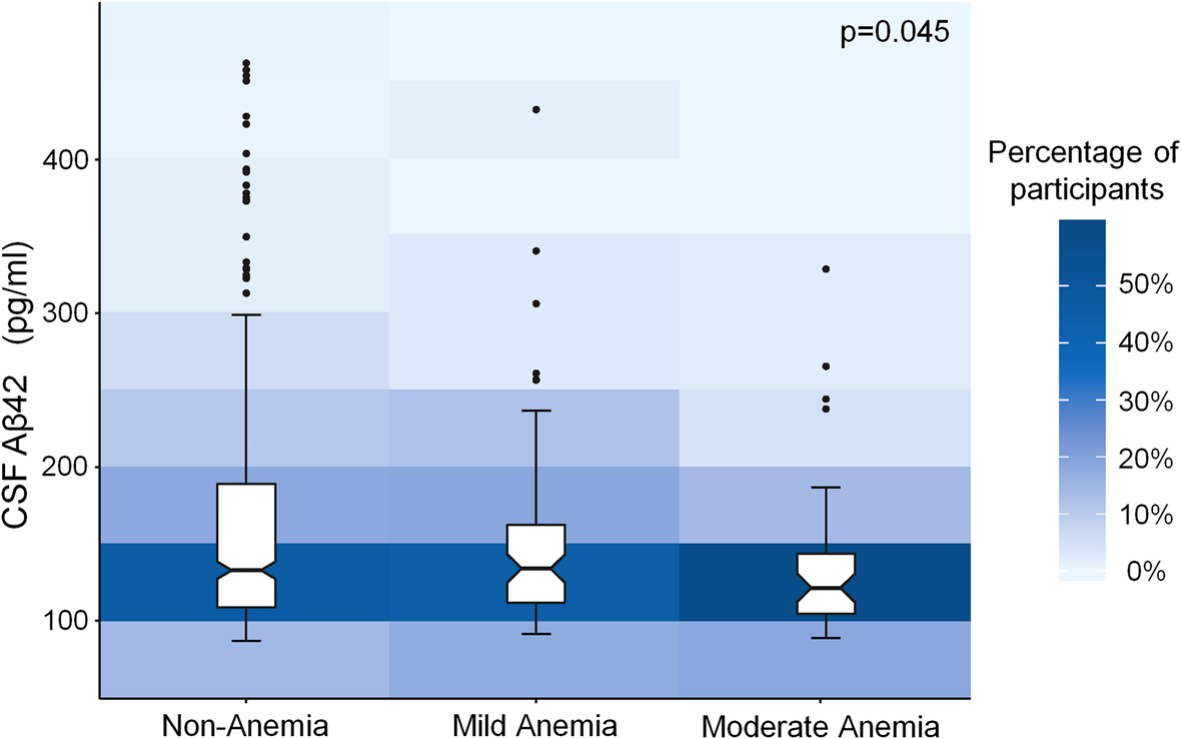
Association between the severity of anaemia and CSF Aβ42. Participants with more severe anaemia had lower CSF Aβ42 levels (*p* = 0.045)

The associations between the severity of anaemia and CSF biomarkers were also investigated. Among 117 anaemia patients in our study, there were 74 participants with mild anaemia, 41 participants with moderate anaemia and 2 participants with severe anaemia, according to the WHO criteria. These two participants with severe anaemia were not included in the analysis due to the small sample size. After adjusting for age, gender, educational levels, *APOE* ε4 alleles, comorbidities (history of coronary heart disease, history of stroke, history of hypertension, history of diabetes mellitus, history of dyslipidaemia), and glomerular filtration rate, patients with more severe anaemia had lower CSF Aβ42 levels (*p* = 0.045, Fig. 2). As expected, no significant associations were found of the severity of anaemia with CSF Aβ40, t-tau, p-tau, t-tau/Aβ42, p-tau/Aβ42 and Aβ42/Aβ40 levels (*p* = 0.505 for CSF Aβ40, *p* = 0.781 for CSF t-tau, *p* = 0.256 for CSF p-tau, *p* = 0.101 for CSF t-tau/Aβ42, *p* = 0.299for CSF p-tau/Aβ42, *p* = 0.166 for CSF Aβ42/Aβ40).

## Discussion

Increasing attention has been paid to environmental risk factors of AD in order to find out effective ways to prevent AD. Many chronic diseases including anaemia were found to be associated with AD risk and may aggravate AD pathology. In this study, we investigated the associations between anaemia and CSF AD biomarkers. Anaemia patients had lower CSF Aβ42 levels than participants without anaemia. Besides, the severity of anaemia was also associated with the levels of CSF Aβ42. A decreasing trend of CSF Aβ42 was found in patients with more severe anaemia. Anaemia and AD are common diseases in the elderly. Our study focuses on cognitively normal individuals, which help us explore that the association of AD and anaemia arises at a pre-clinical stage. These findings supported the close relationships between anaemia and AD risk.

Previous studies showed that low haemoglobin and anaemia were associated with cognitive impairment [13, 14]. Anaemia patients had a higher risk of cognitive impairment with an odds ratio of 1.51 [14]. Besides, a fivefold higher dementia incidence was revealed in severe anaemia patients by a longitudinal study [15]. Although the results were inconsistent, most of the studies showed that anaemia was also associated with AD. Individuals with anaemia were reported to have a higher risk of AD [16, 17]. The diagnostic methods used in previous studies were different, which might result in conflicting findings. Studies on AD biomarkers may provide more convincible evidence. Our results that anaemia patients had lower levels of CSF Aβ42 further confirmed the associations between anaemia and AD biological biomarkers. Furthermore, we revealed an association between the severity of anaemia and CSF Aβ42, which suggested that patients with more severe anaemia might have a greater AD risk. This result was also consistent with a previous study that revealed an increasing trend of dementia incidence in patients with more severe anaemia [15].

Several possible mechanisms underlying how anaemia was involved in AD have been proposed. Long-term reductions in haemoglobin may lead to craniocerebral hypoxic injury. Increasing evidence suggests that hypoxia facilitates the pathogenesis of AD through accelerating the accumulation of Aβ, increasing the hyperphosphorylation of tau [18]. The main finding in our study is an association between presence of anaemia and low CSF Aβ42 but not p-tau or t-tau in cognitively normal participants. Our study may have provided evidence that the amyloid cascade hypothesis assumes seems to be a reasonable pathogenic model. In addition, the lack of vitamin B12 and iron, which is an important reason of anaemia, is also a risk factor of AD [19].

There were some limitations of this study. Our analysis was cross-sectional, therefore limiting causal inferences and raising the possibility that brain changes may precede the development of anaemia. Furthermore, due to the limitation of sample size and the insufficient data, we were unable to analyse the associations of haemoglobin concentration and the anaemia type with CSF AD biomarkers. Additionally, there was no information on the causes of anaemia (for example, studies on vitamin B12, folate, erythropoietin levels, or iron) and a neurodegeneration biomarker such as CSF neurofilament light chain or MRI measures of atrophy in this study.

Our study showed further evidence for the associations of anaemia with CSF AD biomarkers in cognitively normal elders. The treatment of anaemia would be useful in the prevention of AD. Further studies are still necessary to investigate the longitudinal associations between anaemia and the changes in AD biomarkers.

## Conclusion

These results indicated that anaemia might have a detrimental effect on AD core pathology. The treatment for anaemia may be useful in the prevention of AD.

## Supplementary Information

**Additional file 1: Table S1**. Associations between anaemia and AD biomarkers. **Table S2**. Associations between the severity of anaemia and AD biomarkers. **Table S3**. Association of anemia with CSF Aβ42. **Table S4**. Association of anemia with CSF Aβ40. **Table S5**. Association of anemia with CSF t-tau. **Table S6**. Association of anemia with CSF p-tau. **Table S7**. Association of anemia with CSF t-tau/Aβ42 ratio. **Table S8**. Association of anemia with CSF p-tau/Aβ42 ratio. **Table S9**. Association of anemia with CSF Aβ42/Aβ40 ratio. **Table S10**. Associations between the severity of anaemia and CSF Aβ42. **Table S11**. Associations between the severity of anaemia and CSF Aβ40. **Table S12**. Associations between the severity of anaemia and CSF t-tau. **Table S13**. Associations between the severity of anaemia and CSF p-tau. **Table S14**. Associations between the severity of anaemia and t-tau/Aβ42 ratio. **Table S15**. Associations between the severity of anaemia and p-tau/Aβ42 ratio. **Table S16**. Associations between the severity of anaemia and Aβ40/Aβ42 ratio.

## Data Availability

The datasets used and/or analysed during the current study are available from the corresponding author on reasonable request.

## Abbreviations

AD: Alzheimer’s disease
CABLE: Chinese Alzheimer’s Biomarker and Lifestyle Study
CSF: Cerebrospinal fluid
*APOE* ε4: Apolipoprotein E
CM-MMSE: China-Modified Mini-Mental State Examination
Aβ: β-Amyloid
t-tau: Total tau
p-tau: Phosphorylated tau
HGB: Hemoglobin
SD: Standard Deviation
