# Supplementary material for: Anaemia and cerebrospinal fluid biomarkers of Alzheimer’s pathology in cognitively normal elders: the CABLE study
Source: BMC Neurol. 2021 Nov 19;21:454. doi: 10.1186/s12883-021-02487-z (PMC8603492; doi:10.1186/s12883-021-02487-z)
Supplement: Supplementary file 1 — Additional file 1: Table S1. Associations between anaemia and AD biomarkers. Table S2. Associations between the severity of anaemia and AD biomarkers. Table S3. Association of anemia with CSF Aβ42. Table S4. Association of anemia with CSF Aβ40. Table S5. Association of anemia with CSF t-tau. Table S6. Association of anemia with CSF p-tau. Table S7. Association of anemia with CSF t-tau/Aβ42 ratio. Table S8. Association of anemia with CSF p-tau/Aβ42 ratio. Table S9. Association of anemia with CSF Aβ42/Aβ40 ratio. Table S10. Associations between the severity of anaemia and CSF Aβ42. Table S11. Associations between the severity of anaemia and CSF Aβ40. Table S12. Associations between the severity of anaemia and CSF t-tau. Table S13. Associations between the severity of anaemia and CSF p-tau. Table S14. Associations between the severity of anaemia and t-tau/Aβ42 ratio. Table S15. Associations between the severity of anaemia and p-tau/Aβ42 ratio. Table S16. Associations between the severity of anaemia and Aβ40/Aβ42 ratio. [file 12883_2021_2487_MOESM1_ESM.docx]

**Table S1. Associations between anaemia and AD biomarkers**

|  |  | **Model 1** | |  | **Model 2** | |
| --- | --- | --- | --- | --- | --- | --- |
|  |  | **β** | **P value** |  | **β** | **P value** |
| CSF Aβ42 |  | -15.06 | **0.035** |  | -15.54 | **0.035** |
| CSF Aβ40 |  | -63.01 | 0.814 |  | -69.85 | 0.799 |
| CSF t-tau |  | 4.25 | 0.560 |  | 1.51 | 0.838 |
| CSF p-tau |  | -1.21 | 0.162 |  | -1.15 | 0.197 |
| T-tau/Aβ42 ratio |  | 0.13 | 0.050 |  | 0.10 | 0.117 |
| P-tau/Aβ42 ratio |  | 0.009 | 0.316 |  | 0.008 | 0.397 |
| Aβ42/ Aβ40 ratio |  | -4.129e-03 | 0.103 |  | -4.12e-03 | 0.097 |

Model 1: adjusted for age, gender, education years, *APOE* ε4 alleles;

Model 2：Model 1 +comorbidities (history of coronary heart disease, history of stroke, hypertension, diabetes mellitus, dyslipidaemia) + glomerular filtration rate.

**Table S2. Associations between the severity of anaemia and AD biomarkers**

|  |  | **Model 1** | |  | **Model 2** | |
| --- | --- | --- | --- | --- | --- | --- |
|  |  | **β** | **P value** |  | **β** | **P value** |
| CSF Aβ42 |  | 9.76 | **0.045** |  | 10.08 | **0.045** |
| CSF Aβ40 |  | 100.98 | 0.579 |  | 25.58 | 0.505 |
| CSF t-tau |  | -4.351 | 0.380 |  | 1.42 | 0.781 |
| CSF p-tau |  | 0.707 | 0.231 |  | 0.69 | 0.256 |
| T-tau/Aβ42 ratio |  | -0.101 | **0.027** |  | 0.07 | 0.101 |
| P-tau/Aβ42 ratio |  | -0.008 | 0.218 |  | 0.007 | 0.299 |
| Aβ42/ Aβ40 ratio |  | 0.000 | 0.163 |  | 0.002 | 0.166 |

**Table S3. Association of anemia with CSF Aβ42**

|  |  | **β (95% CI)** | **P value** |
| --- | --- | --- | --- |
| Age |  | 0.051 (-0.64, 0.74) | 0.884 |
| Gender |  | 11.853 (0.77, 22.93) | 0.036 |
| *APOE* ε4 alleles |  | -15.454 (-28.97, -1.93) | 0.025 |
| Education years |  | -0.156 (-1.42, 1.11) | 0.808 |
| Coronary heart disease |  | 7.997 (-9.38, 25.38) | 0.366 |
| History of stroke |  | 39.431 (4.63, 74.22) | 0.026 |
| Hypertension |  | -1.368 (-12.95, 10.21) | 0.816 |
| Diabetes mellitus |  | 7.235 (-8.49, 22.96) | 0.366 |
| Glomerular filtration rate |  | -0.040 (-0.49, 0.40) | 0.858 |
| Dyslipidemia |  | 23.825 (-5.29, 52.94) | 0.108 |
| CSF Aβ42 |  | -15.541 (-29.96, -1.12) | 0.034 |

CI = confidence interval

Adjusted for age, gender, education years, *APOE* ε4 alleles, comorbidities (history of coronary heart disease, history of stroke, hypertension, diabetes mellitus, dyslipidemia), glomerular filtration rate.

**Table S4. Association of anemia with CSF Aβ40**

|  |  | **β (95% CI)** | **P value** |
| --- | --- | --- | --- |
| Age |  | 22.19 (-3.82, 48.20) | 0.094 |
| Gender |  | -204.85 (-620.13, 210.42) | 0.333 |
| *APOE* ε4 alleles |  | 46.14 (-460.58, 552.87) | 0.858 |
| Education years |  | -22.66 (-70.12, 24.80) | 0.348 |
| Coronary heart disease |  | 468.43 (-1119.97,183.12) | 0.158 |
| History of stroke |  | -517.58 (-1821.58,786.43) | 0.436 |
| Hypertension |  | 310.92 (-123.32, 745.15) | 0.160 |
| Diabetes mellitus |  | 90.29 (-499.13,679.72) | 0.763 |
| Glomerular filtration rate |  | -14.70 (-31.56, 2.17) | 0.087 |
| Dyslipidemia |  | 1439.39 (348.12, 2530.66) | 0.009 |
| CSF Aβ40 |  | -69.85 (-610.30, 470.59) | 0.799 |

**Table S5. Association of anemia with CSF t-tau**

|  |  | **β (95% CI)** | **P value** |
| --- | --- | --- | --- |
| Age |  | 1.23 (0.53, 1.94) | <0.001 |
| Gender |  | -1.51 (-12.76, 9.73) | 0.791 |
| *APOE* ε4 alleles |  | 5.25 (-8.47, 18.97) | 0.452 |
| Education years |  | -0.59 (-1.87, 0.69) | 0.364 |
| Coronary heart disease |  | 8.00 (-25.65,9.64) | 0.373 |
| History of stroke |  | 6.57 (-28.74, 41.89) | 0.714 |
| Hypertension |  | 1.64 (-0.11, 23.40) | 0.052 |
| Diabetes mellitus |  | 1.77 (-17.74, 14.18) | 0.827 |
| Glomerular filtration rate |  | 0.24 (-0.70, 0.20) | 0.288 |
| Dyslipidemia |  | 18.17 (-11.38, 47.72) | 0.227 |
| CSF t-tau |  | 1.51 (-13.12, 16.15) | 0.838 |

**Table S6. Association of anemia with CSF p-tau**

|  |  | **β (95% CI)** | **P value** |
| --- | --- | --- | --- |
| Age |  | 0.12 (0.03,0.20) | 0.005 |
| Gender |  | 0.08 (-1.27,1.43) | 0.906 |
| *APOE* ε4 alleles |  | 0.86 (-0.78, 2.51) | 0.305 |
| Education years |  | -0.15 (-0.30, 0.002) | 0.053 |
| Coronary heart disease |  | 1.13 (-0.98,3.25) | 0.293 |
| History of stroke |  | 0.30 (-3.93, 4.55) | 0.886 |
| Hypertension |  | 1.14 (-0.27,2.55) | 0.112 |
| Diabetes mellitus |  | 1.17 (-3.09, 0.73) | 0.227 |
| Glomerular filtration rate |  | 0.03 (-0.08, 0.02) | 0.220 |
| Dyslipidemia |  | 0.76 (-2.78, 4.31) | 0.671 |
| CSF p-tau |  | -1.15 (-2.91, 0.60) | 0.197 |

**Table S7. Association of anemia with CSF t-tau/Aβ42 ratio**

|  |  | **β (95% CI)** | **P value** |
| --- | --- | --- | --- |
| Age |  | 0.007 (0.0007,0.013) | 0.028 |
| Gender |  | -0.08 (-0.19,0.01) | 0.100 |
| *APOE* ε4 alleles |  | 0.16 (0.04,0.29) | 0.009 |
| Education years |  | -0.004 (-0.01, 0.007) | 0.451 |
| Coronary heart disease |  | 0.09 (-0.25,0.06) | 0.249 |
| History of stroke |  | -0.09 (-0.42, 0.23) | 0.568 |
| Hypertension |  | 0.10 (-0.007, 0.21) | 0.066 |
| Diabetes mellitus |  | 0.09 (-0.24, 0.04) | 0.185 |
| Glomerular filtration rate |  | 0.002 (-0.006, 0.001) | 0.201 |
| Dyslipidemia |  | -0.04 (-0.31, 0.22) | 0.753 |
| CSF t-tau/Aβ42 ratio |  | 0.10 (-0.02,0.24) | 0.117 |

**Table S8. Association of anemia with CSF p-tau/Aβ42 ratio**

|  |  | **β (95% CI)** | **P value** |
| --- | --- | --- | --- |
| Age |  | 0.0006 (-0.0002, 0.001) | 0.174 |
| Gender |  | -0.01 (-0.03, -0.001) | 0.028 |
| *APOE* ε4 alleles |  | 0.03 (0.013, 0.05) | <0.001 |
| Education years |  | -0.0009 (-0.002, 0.0008) | 0.308 |
| Coronary heart disease |  | 0.003 (-0.02, 0.02) | 0.771 |
| History of stroke |  | -0.02 (-0.07. 0.02) | 0.276 |
| Hypertension |  | 0.01 (-0.004, 0.02) | 0.145 |
| Diabetes mellitus |  | 0.02 (-0.042, 0.0006) | 0.057 |
| Glomerular filtration rate |  | 0.0004 (-0.001, 0.0001) | 0.124 |
| Dyslipidemia |  | -0.03 (-0.07, 0.01) | 0.144 |
| CSF p-tau/Aβ42 ratio |  | 0.008 (-0.01, 0.02) | 0.397 |

**Table S9. Association of anemia with CSF Aβ42/Aβ40 ratio**

|  |  | **β (95% CI)** | **P value** |
| --- | --- | --- | --- |
| Age |  | -1.10e-04 (-0.0002, 0.001) | 0.358 |
| Gender |  | 3.26e-03 (-0.03, -0.001) | 0.088 |
| *APOE* ε4 alleles |  | -3.94e-03 (0.013, 0.05) | 0.091 |
| Education years |  | 2.37e-04 (-0.002, 0.0008) | 0.277 |
| Coronary heart disease |  | 4.61e-03 (-0.02, 0.02) | 0.185 |
| History of stroke |  | 7.96e-03 (-0.07. 0.02) | 0.319 |
| Hypertension |  | -1.99e-03 (-0.004, 0.02) | 0.507 |
| Diabetes mellitus |  | 1.79e-03 (-0.042, 0.0006) | 0.124 |
| Glomerular filtration rate |  | 3.58e-05 (-0.001, 0.0001) | 0.644 |
| Dyslipidemia |  | -5.08e-03 (-0.07, 0.01) | 0.311 |
| CSF Aβ42/Aβ40 ratio |  | -4.12e-03 (-0.01, 0.02) | 0.097 |

**Table S10. Associations between the severity of anaemia and CSF Aβ42**

|  |  | **β (95% CI)** | **P value** |
| --- | --- | --- | --- |
| Age |  | 0.02 (0.66,0.72) | 0.934 |
| Gender |  | 11.99 (0.91, 23.07) | 0.033 |
| *APOE* ε4 alleles |  | 15.40 (-28.94, -1.87) | 0.025 |
| Education years |  | -0.13 (-1.40, 1.13) | 0.832 |
| Coronary heart disease |  | 8.05 (-9.33,25.44) | 0.363 |
| History of stroke |  | 38.74 (3.97, 73.52) | 0.029 |
| Hypertension |  | 1.33 (-12.92, 10.25) | 0.821 |
| Diabetes mellitus |  | 7.07 (-8.65,22.80) | 0.377 |
| Glomerular filtration rate |  | 0.05 (-0.50, 0.40) | 0.821 |
| Dyslipidemia |  | 24.02 (-5.09,53.14) | 0.105 |
| CSF Aβ42 |  | 10.08 (0.20, 19.97) | 0.045 |

**Table S11. Associations between the severity of anaemia and CSF Aβ40**

|  |  | **β (95% CI)** | **P value** |
| --- | --- | --- | --- |
| Age |  | 22.18 (-3.80, 48.17) | 0.094 |
| Gender |  | 211.56 (-626.45,203.31) | 0.317 |
| *APOE* ε4 alleles |  | 31.44 (-475.39,538.28) | 0.903 |
| Education years |  | -22.61(-70.06, 24.82) | 0.349 |
| Coronary heart disease |  | 71.25 (-1122.58,180.06) | 0.155 |
| History of stroke |  | 504.32 (-1806.63, 797.99) | 0.447 |
| Hypertension |  | 06.812 (-127.28, 740.90) | 0.165 |
| Diabetes mellitus |  | 97.64 (-491.36, 686.66) | 0.7449 |
| Glomerular filtration rate |  | 15.511 (-32.48, 1.46) | 0.073 |
| Dyslipidemia |  | 426.46 (335.73, 2517.19) | 0.010 |
| CSF Aβ40 |  | 25.58 (-244.64, 495.81) | 0.505 |

**Table S12. Associations between the severity of anaemia and CSF t-tau**

|  |  | **β (95% CI)** | **P value** |
| --- | --- | --- | --- |
| Age |  | 1.23 (0.53,1.94) | < 0.001 |
| Gender |  | -1.48 (-12.72, 9.75) | 0.794 |
| *APOE* ε4 alleles |  | 5.32 (-8.40, 19.06) | 0.446 |
| Education years |  | -0.59 (-1.88, 0.68) | 0.363 |
| Coronary heart disease |  | 7.99 (-25.63, 9.65) | 0.374 |
| History of stroke |  | 6.55 (-28.73, 41.83) | 0.715 |
| Hypertension |  | 1.66 (-0.09, 23.42) | 0.051 |
| Diabetes mellitus |  | 1.80 (-17.76, 14.15) | 0.824 |
| Glomerular filtration rate |  | 0.24 (-0.70, 0.21) | 0.302 |
| Dyslipidemia |  | 18.22 (-11.32, 47.77) | 0.226 |
| CSF t-tau |  | 1.42 (-11.45, 8.61) | 0.781 |

**Table S13. Associations between the severity of anaemia and CSF p-tau**

|  |  | **β (95% CI)** | **P value** |
| --- | --- | --- | --- |
| Age |  | 0.11 (0.03, 0.20) | 0.005 |
| Gender |  | 0.09 (-1.25, 1.44) | 0.888 |
| *APOE* ε4 alleles |  | 0.87 (-0.77, 2.52) | 0.298 |
| Education years |  | -0.15 (-0.30, 0.003) | 0.055 |
| Coronary heart disease |  | 1.14 (-0.97, 3.26) | 0.291 |
| History of stroke |  | 0.24 (-3.99, 4.48) | 0.909 |
| Hypertension |  | 1.14 (-0.26, 2.56) | 0.111 |
| Diabetes mellitus |  | 1.19 (-3.11, 0.72) | 0.221 |
| Glomerular filtration rate |  | 0.03 (-0.08, 0.02) | 0.219 |
| Dyslipidemia |  | 0.79 (-2.76, 4.34) | 0.662 |
| CSF p-tau |  | 0.69 (-0.50, 1.90) | 0.256 |

**Table S14. Associations between the severity of anaemia and t-tau/Aβ42 ratio**

|  |  | **β(95% CI)** | **P value** |
| --- | --- | --- | --- |
| Age |  | 0.007 (0.0009, 0.01) | 0.025 |
| Gender |  | -0.08 (-0.19, 0.01) | 0.098 |
| *APOE* ε4 alleles |  | 0.16 (0.04, 0.29) | 0.009 |
| Education years |  | -0.004 (-0.01, 0.007) | 0.437 |
| Coronary heart disease |  | 0.09 (-0.25, 0.06) | 0.249 |
| History of stroke |  | -0.09 (-0.41, 0.23) | 0.581 |
| Hypertension |  | 0.10 (-0.007, 0.21) | 0.066 |
| Diabetes mellitus |  | 0.09 (-0.24, 0.04) | 0.187 |
| Glomerular filtration rate |  | 0.002 (-0.006, 0.001) | 0.229 |
| Dyslipidemia |  | -0.04 (-0.31, 0.22) | 0.752 |
| CSF t-tau/Aβ42 ratio |  | 0.07 (-0.17, 0.01) | 0.101 |

**Table S15. Associations between the severity of anaemia and p-tau/Aβ42 ratio**

|  |  | **β (95% CI)** | **P value** |
| --- | --- | --- | --- |
| Age |  | 0.0006 (-0.0002, 0.001) | 0.167 |
| Gender |  | -0.01 (-0.03, -0.001) | 0.028 |
| *APOE* ε4 alleles |  | 0.032 (0.01, 0.05) | < 0.001 |
| Education years |  | -0.0009 (-0.002, 0.0008) | 0.302 |
| Coronary heart disease |  | 0.003 (-0.02, 0.02) | 0.773 |
| History of stroke |  | -0.02 (-0.07, 0.02) | 0.276 |
| Hypertension |  | 0.01 (-0.004, 0.02) | 0.142 |
| Diabetes mellitus |  | 0.02 (-0.04, 0.0005) | 0.056 |
| Glomerular filtration rate |  | 0.0004 (-0.001, 0.0001) | 0.145 |
| Dyslipidemia |  | -0.02 (-0.07, 0.01) | 0.146 |
| CSF p-tau/Aβ42 ratio |  | 0.007 (-0.02, 0.006) | 0.299 |

**Table S16. Associations between the severity of anaemia and Aβ40/Aβ42 ratio**

|  |  | **β (95% CI)** | **P value** |
| --- | --- | --- | --- |
| Age |  | -1.16e-04 (-0.0003, 0.0001) | 0.331 |
| Gender |  | 3.33e-03 (-0.0004, 0.007) | 0.081 |
| *APOE* ε4 alleles |  | -3.87e-03(-0.008, 0.0007) | 0.097 |
| Education years |  | 2.43e-04 (-0.0001, 0.0006) | 0.266 |
| Coronary heart disease |  | 4.64e-03 (-0.001, 0.01) | 0.122 |
| History of stroke |  | 7.71e-03 (-0.004, 0.01) | 0.198 |
| Hypertension |  | -1.96e-03 (-0.005, 0.001) | 0.326 |
| Diabetes mellitus |  | 1.72e-03 (-0.003, 0.007) | 0.525 |
| Glomerular filtration rate |  | 3.58e-05 (-0.0001,0.0001) | 0.646 |
| Dyslipidemia |  | -4.97e-03 (-0.01,0.004) | 0.322 |
| CSF Aβ40/Aβ42 ratio |  | 2.36e-03 (-0.0009, 0.005) | 0.166 |
